# Exploring parents’ perspectives on health technology to support communication in home-based pediatric palliative care: a qualitative study

**DOI:** 10.1186/s12904-026-02006-2

**Published:** 2026-01-29

**Authors:** Linda J. Martinsen, Heidi Holmen, Simen A. Steindal, Anette Winger

**Affiliations:** 1 Department of Nursing and Health Promotion, Faculty of Health Sciences, Oslo Metropolitan University, Postbox 4, St. Olavs Place, Oslo, NO-0130 Norway; 2 VID Specialized University, Oslo, Norway; 3 Oslo University Hospital, Oslo, Norway; 4 Lovisenberg Diaconal University College, Oslo, Norway

**Keywords:** Communication, Health technology, Home-based care, Pediatric palliative care, Qualitative study

## Abstract

**Background:**

Children eligible for palliative care represent a highly diverse population, making comprehensive, family-centered approaches essential. As many families value being at home and maintaining their daily activities and routines, healthcare services need to be tailored to each family’s unique circumstances. Health technology offers promising support for home-based pediatric palliative care and has been suggested to enhance communication, coordination, and continuity of care. Despite growing interest, a significant research gap regarding the systematic integration of health technology into pediatric palliative care, particularly in understanding how digital tools can be meaningfully embedded into everyday life. Given this research gap, this study aimed to explore parents’ perspectives regarding the use of health technology in home-based pediatric palliative care, with a particular focus on facilitating communication with healthcare professionals.

**Methods:**

This qualitative exploratory study was conducted with five focus groups comprising 18 Norwegian parents of children aged 1.5–18 years old with life-threatening or life-limiting conditions. Data were analyzed using reflexive thematic analysis.

**Results:**

Three themes were created in the analysis: (1) Digital consultations as tools for reducing disruptions to everyday life; (2) Who needs to know what? Balancing access and security in electronic health records; and (3) Keep the health technology simple.

**Conclusion:**

Health technology has the potential to improve flexibility, reduce travel burdens, and support everyday life for families in which a child is receiving home-based pediatric palliative care. Its effectiveness depends on usability, integration with healthcare systems, and the parents’ ability to control access to their child’s health information. Ensuring both accessibility and security is essential for building trust in health technology. Successful implementation of health technology requires co-design with users and stakeholders to create sustainable solutions that meet the complex needs of children receiving home-based palliative care.

**Supplementary Information:**

The online version contains supplementary material available at 10.1186/s12904-026-02006-2.

## Background

Pediatric palliative care (PPC) is a comprehensive approach to children with life-limiting or life-threatening conditions, aiming to improve quality of life by supporting the needs of both the child and their family [1]. Being at home while receiving PPC would allow children to remain in familiar surroundings and maintain their daily lives as normally as possible with their families, thereby reducing parents’ stress and anxiety [2, 3]. To succeed with home-based PPC, parents require reliable communication and support from healthcare professionals, which are essential for coordinating services and providing practical assistance to both the child and the family [4, 5]. However, in home-based PPC, families and healthcare professionals often face various persistent challenges in accessing information and coordinating care, leading to gaps in service delivery [6, 7]. Effective communication between families and healthcare professionals, though essential, is often insufficient, leading to misunderstandings and unmet expectations for parents [3, 6]. Furthermore, there can be failure in the coordination of care among different healthcare professionals, resulting in inconsistencies in the child’s follow-up and how home-based PPC is delivered [8, 9]. Healthcare professionals also encounter difficulties in providing comprehensive and coordinated home-based care due to time constraints, a lack of specialized training, and insufficient resources [7, 10, 11].

Precise estimates of the number of children with PPC needs in Norway are lacking. However, applying a prevalence estimate of 66 per 10,000 children for life-limiting or life-threatening conditions suggests that approximately 8,000 children nationwide live with these conditions [12, 13]. Home-based PPC in Norway is understood as a broad concept, encompassing medical procedures, training and guidance for caregivers and healthcare professionals, as well as psychosocial support. Home-based PPC should be tailored to the individual family’s needs and may be relevant across all phases of the illness trajectory [1, 14]. Norwegian health authorities recommend that primary and specialist health services facilitate the provision of care for children with life-limiting or life-threatening conditions at home and in their local environment, as far as possible [14, 15]. Delivery of home-based PPC in Norway varies but it generally aligns with international standards [12]. This typically involves support from the child’s current care team, which includes healthcare professionals from both primary and specialist healthcare. Primary care providers may consist of physiotherapists, occupational therapists, general practitioners, public health nurses and children’s coordinators. Specialist healthcare support often comes from the General Pediatric Department and Children’s Habilitation Department for Children and Adolescents. The Habilitation Department for Children and Adolescents is a healthcare service in Norway that provides assessment, treatment, and follow-up for children and young people with congenital or early-acquired disabilities of a complex and long-term nature (e.g. life-limiting and life-threatening conditions). The service works in an interdisciplinary manner to promote optimal functioning, coping, and quality of life for the child and their family. Children covered by the service live at home and receive follow-up care on an outpatient basis or within their local community, rather than as inpatients [16]. Home-based PPC can be supplemented by advanced hospital at home services or hospital-based PPC teams, who work alongside and/or together with both primary and specialist healthcare professionals. While some families maintain regular contact with home-based PPC teams offering 24/7 digital and/or home visits, others receive assistance primarily during complex treatment phases or at end-of-life, with care tailored to individual needs [13, 17].

One promising way to improve communication and coordination for parents and healthcare professionals when a child is receiving PPC at home is through the use of health technology [18, 19]. In the present study, health technology is defined as information and communication technologies used for a broad range of applications to enhance patient-centered care. Health technology encompasses a broad understanding and includes electronic health (eHealth), telehealth, telemedicine, mobile health (mHealth), and digital health [20]. Previous studies indicate that health technology has the potential to improve home-based PPC through tools for remote monitoring, digital consultations, and real-time communication between parents and healthcare professionals [18, 21]. These advances could enable timely interventions and comprehensive support, thus supporting the quality of life of children with life-limiting or life-threatening conditions within the comfort and familiarity of their home environment [18, 21]. To facilitate the successful use of health technology in home-based PPC, it is important to understand the conditions under which such care is delivered and the needs of those requiring it [18, 22].

In this study, to better understand parents’ perceptions of health technology for communication in the context of PPC, the unified theory of acceptance and use of technology (UTAUT) was applied to discuss our findings [23]. UTAUT allows for a deeper exploration of the conditions under which health technology is perceived as meaningful and useful in the context of home-based PPC. This theory identifies four key factors that influence this process. *Performance expectancy* refers to the degree to which a person believes that using a particular technology will help them achieve desired outcomes. *Effort expectancy* captures how easy or difficult technology is perceived to be; the simpler it is to use, the more likely individuals are to adopt it. *Social influence* reflects the extent to which people feel that important others, such as healthcare professionals, think they as parents should use technology, highlighting the role of social pressure and norms. Finally, *facilitating conditions* pertains to the availability of resources and support, such as training, infrastructure, and technical assistance, which enable individuals to effectively use the technology [24].

In the literature, there are limited explorations of end-user perspectives from parents regarding the use of health technology in home-based PPC [18]. There is also a need to explore parents’ perspectives on health technology for home-based PPC, representing children with various conditions and in different phases of PPC. A large proportion of existing research focuses on home-based care during the end-of-life phase [25, 26], even though most children in palliative care live with their condition for an extended period [1]. New perspectives could enhance our understanding of the breadth and diversity of those requiring PPC, ensuring more effective and comprehensive home-based care for these families [18, 21]. Against this background, we aimed to explore Norwegian parents’ perspectives on the use of health technology in home-based PPC, with a particular focus on facilitating communication with healthcare professionals.

## Methodology

### Study design

Our study employed a qualitative exploratory design, using focus groups to explore the perspectives of parents of children with life-limiting or life-threatening conditions regarding the use of health technology in home-based PPC in Norway. We chose to employ focus groups because of the additional insights potentially provided from the interactions and discussions among the participants, compared with those obtained through individual interviews [27]. This method was particularly appropriate as we aimed to explore the shared perspectives of the participants on topics they could all relate to [27]. This study was reported in accordance with the Reflexive Thematic Analysis Reporting Guidelines (RTARG) [28]. The data presented here are part of a larger dataset obtained through the research project Children in Palliative Care – Health Technology in Home-based Pediatric Palliative Care (CHIP homeTec), which explores the utilization of health technology in home-based PPC. The focus groups in this study explored topics related to the family’s needs for home-based care in general and their perceptions of the use of health technology to address these needs. While parents’ reflections on home-based PPC in general have already been reported elsewhere [17], their perceptions of the use of health technology to address their needs are further explored in this article.

### Recruitment and participants

Parents of children living with life-limiting or life-threatening conditions were recruited through a digital survey conducted as part of the CHIP homeTec project, where they accepted being contacted for more information regarding potential participation in the above-mentioned focus groups. The participants were recruited through a leaflet posted at an outpatient clinic for children, including those receiving home-based PPC, for inclusion in a pilot focus group. We used purposeful sampling according to the following inclusion criteria: parents of children aged 0–18 years with a life-limiting or life-threatening condition, living in Norway, and able to participate in a Norwegian-speaking focus group. Participants opting to receive more details were contacted by the first author (L.J.M.) by e-mail or phone to provide written and oral information about the study. A total of 25 parents consented to participate. However, six parents were eventually unable to participate due to their own or a family member’s acute illness or some other unforeseen issue. In addition, one parent withdrew from the study, the reason for this was not given.

The final study participants comprised 18 parents, representing 14 children with various life-limiting or life-threatening conditions. The median age of the parents was 40.0 years (range: 29.0–49.0), and 15 of the 18 were female. The children had a median age of 5.8 years (range: 1.5–18.0), and 8 of the 14 were females. Additionally, 11 of the children had siblings. In terms of the conditions necessitating PPC [1], 3 of the children had life-threatening conditions, 4 had progressive conditions for which there were no curative treatment options, and 5 had irreversible but nonprogressive conditions. Furthermore, 2 children had other combined conditions necessitating PPC. The participants were distributed across Norway’s four Regional Health Authority (RHA) regions: 4 from Northern Norway RHA, 1 from Central Norway RHA, 7 from Western Norway RHA, and 2 from South-Eastern Norway RHA.

### Dataset generation

A semi-structured interview guide was developed to facilitate reflection on the topic of the study and provide a focus for the discussions within the focus groups. A reference group, including user representatives, healthcare professionals, and other researchers engaged in the field of PPC, reviewed the interview guide to ensure that the questions were well formulated and relevant. The pilot testing of the interview guide within the focus group led to minor adjustments in wording and in the order of the questions, but no questions were removed or added to the final interview guide. As a result, the pilot interview was included as part of the study’s data collection. The interview guide included open-ended and probing questions regarding parents’ perspectives on healthcare services, home-based care, and health technology. It also addressed the everyday needs of the parents, the ill child, and their siblings, as well as how to meet those needs effectively, with or without health technology. This study focuses specifically on the questions regarding the parents’ perspectives on the use of health technology in home-based PPC.

Five focus groups, including the pilot one, were conducted between December 2023 and March 2024. Each focus group consisted of 3–5 parents. In some groups, both parents of a child participated, while in others, either the mother or the father attended alone. Each focus group lasted from about 50 to 85 min (mean: 65 min), with no breaks or interruptions. All focus groups were audio-recorded digitally, and the data were encrypted and transferred to secure storage. Three focus groups were conducted in a meeting room outside healthcare settings, while two were conducted online using Microsoft Teams with both audio and video recording. The first author (L.J.M.) functioned as the moderator in all groups, while one of the co-authors (A.W. or S.A.S.) acted as the assistant moderator. At the beginning of each focus group, the moderators introduced themselves along with their professional backgrounds and experiences. Participants were informed about CHIP homeTec and the objectives of the project, followed by specific information about the focus group and its aim to explore the use of health technology and home-based PPC.

### Data analysis

**Fig. 1:**
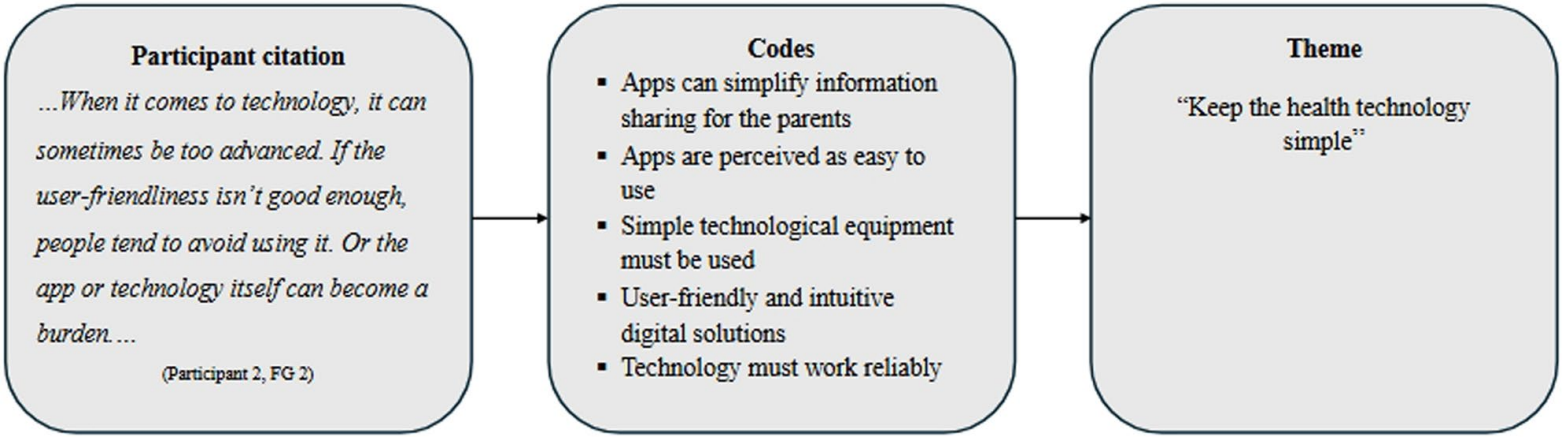
Example of the analytical process and development of themes. Illustrated here is the theme *“Keep the health technology simple”*

The recorded data from the focus groups were transcribed by the first author (L.J.M.). NVivo software (QSR International Pty Ltd., Victoria, Australia) was used to organize and manage the data. The transcripts were analyzed using reflexive thematic analysis [29]. The analysis was conducted by L.J.M. in collaboration with the three co-authors, who were actively making use of their backgrounds and experience and engaging in ongoing discussions throughout all phases of the analytical process. In the first phase of the analysis, the interview transcripts were repeatedly read to become familiar with the dataset, and notes on ideas or thoughts were written directly on printed copies of the transcripts. In the next phase, systematic inductive coding with a semantic approach was initiated to identify patterns from the dataset relevant to the aim of the study. In the third phase, initial themes were generated by grouping codes using NVivo based on identified patterns across the dataset. In the subsequent phase, the candidate themes were reorganized, expanded, or narrowed down through several reviews. The final themes were developed collaboratively by all the authors, who engaged in reflective discussions to explore various interpretations, ensuring that the themes were not only clarified but also accurately named. This collaborative process fostered a wider understanding and enriched the overall analysis, allowing for a comprehensive representation of the participants’ perspectives and naming of the final themes. At the end of the analytical process, the results were documented and supported by the participants’ excerpts (see Fig. 1 for an illustration of the analytical process and the development of themes).

### Preunderstanding

All the authors have a background as healthcare professionals with relevant experience of clinical practice, and their prior knowledge and experience of PPC have influenced their understanding in this study. L.J.M. is a pediatric occupational therapist with 15 years of experience working in the Habilitation Department for Children and Adolescents within the specialized health service. This also includes experience working with children with a wide variety of life-limiting or life-threatening conditions, as well as supporting their families. It also involves performing various types of digital consultations with parents and communicating digitally with other healthcare professionals. This experience has enhanced the understanding of how health technology is applied in PPC from a healthcare professional’s perspective. H.H., S.A.S., and A.W. have clinical experience and/or experience in qualitative research in pediatrics, palliative care, and critically ill patients, as well as in health technology. All authors are members of the Children in Palliative Care (CHIP) research network and are engaged in the research project CHIP homeTec. All the authors actively sought to include diverse perspectives from each other and reflected on how any preunderstanding might affect the research process.

### Ethical considerations

Ethical approval for this study was obtained from the Regional Committees for Medical and Health Research Ethics in Norway (reference number: 251065) and Sikt - Norwegian Agency for Shared Services in Education and Research (reference number: 657413). All the participants received oral and written information about the aim of the study and the possible benefits and consequences of participating. They were informed that participation was voluntary and that they could withdraw at any time. They were also informed that all personal data would be handled confidentially. Before participating in the focus groups, digital informed consent was obtained from all participants. The interviews were recorded, and the data were digitally transferred from the Service for Sensitive Data (TSD) “Diktafon” app to TSD for storage, transcription, and analysis.

## Results

Three themes were created in the analysis: (1) Digital consultations as tools for reducing disruptions to everyday life; (2) Who needs to know what? Balancing access and security in electronic health records; and (3) Keep the health technology simple.

### Digital consultations as tools for reducing disruptions to everyday life

Parents described the experience of traveling to hospitals and healthcare consultations with a child who has a life-limiting or life-threatening condition as both logistically and emotionally challenging. This was true regardless of the distance between home and hospital. In-person hospital visits were not always perceived as necessary by parents, who identified various advantages of digital consultations conducted online, via video or phone. These included reduced time and effort for both the child and the parents. Parents valued having follow-ups as close to their home as possible and expressed a desire to reduce the number of hospital visits for the child. Moreover, parents emphasized that digital consultations contributed to more efficient use of healthcare resources by shortening the time used in consultations. This approach was also seen as potentially reducing costs for the healthcare system through reduced travel expenses and the more effective use of healthcare professionals’ time.

The parents stated that digital consultations could reduce the burden caused by changes in the child’s daily routines, such as being away from school or kindergarten. Additionally, parents noted that digital consultations would be sufficient when a physical examination of the child was not required. For example, meetings with a healthcare professional to assist with applications for social benefits or adjustments to medication and nutrition could be conducted digitally, thereby eliminating the need for in-person attendance with the child.

*We’ve started doing phone consultations with the urology department because*,* quite simply*,* they don’t need to see the child in person. The most important thing is that we talk about how things have gone since the last time and how we should proceed. I keep track of various statistics and measurements and reports on how things have been going. A lot of time is saved by doing this over the phone and it works really well. And the child doesn’t have to interrupt their day or be taken out of kindergarten. Even though we live close to the hospital*,* a visit still takes up half a day of his time in kindergarten* (P2, FG 5).

Another advantage was that digital consultations made communication with healthcare professionals more specific and tailored by focusing on specific needs without meetings being experienced as unnecessary.

*It’s one phone call*,* and we spend three minutes adjusting the settings on the pump*,* and then we’re done… It saves us a lot of time* (P1 and 2, FG 2).

Although parents emphasized many benefits of using health technology, such as improving communication with healthcare professionals, they emphasized the necessity of in-person consultations at the hospital or meetings with other healthcare-related services. Parents also highlighted the importance of the same professionals meeting the child and the parents in person to build and maintain trust and understanding, and to contribute to securing continuity of care.

### Who needs to know what? Balancing access and security in electronic health records

As digital health systems evolve, questions of access, control, and data security have become increasingly prominent for families with a child who require PPC. The parents explained that digital access and oversight facilitated the sharing of information with healthcare professionals and coordinating their child’s follow-up. They expressed a need for the child’s various health-related documents to be consolidated into one shared electronic health record; however, they also emphasized the importance of them maintaining control over the information shared about their child. Parents also wanted control over who had access to their child’s information, expressing concerns regarding hacking and security for their child’s sensitive health information. Some parents worried that leaking or unauthorized sharing of health information would affect factors such as the child’s ability to obtain insurance. The parents were also concerned that health information about the child could be used for purposes besides those for which it is intended.

*But I’m also a bit hesitant about who really needs to know everything. I’m not entirely comfortable with the municipality or the allocation office… I feel they don’t need to know everything about my son. I’ve experienced that they sometimes use that information in ways I don’t think are appropriate. At times*,* they use it against its intended purpose*,* rather than in support of it. So that’s also a factor: how many people actually need access to different types of information about our child* (P1, FG 3).

To ensure that information sharing was more controlled and secure, one parent suggested that an electronic health record with categorized folders, for example, could support the granting of partial access to others. By managing the information, the parents could allow others access for limited periods of time or for specific purposes to share, view, and download files from the folders, a solution that would secure parents’ overall control and oversight over their child’s health information.

In Norway, parents can digitally access their child’s healthcare information through a national electronic health record, “Helsenorge,” using BankID to log in. Parents with registered parental responsibility can view appointments, renew prescriptions and, in certain cases, review medical record notes on behalf of the child. Access to “Helsenorge” posed significant challenges for the parents, particularly when their child turned 12 and 16 years old, because parental access to their children’s electronic health records became increasingly limited due to Norwegian privacy restrictions. Access varies by age, with full access until age 12, partial access from 12 to 16, and no access after 16 unless the child consents. These restrictions led to a loss of oversight and control over crucial information, such as hospital appointments, medical reports, and prescriptions. This shift created additional burdens for parents, who were still responsible for managing their child’s healthcare but were no longer granted the necessary access to do so effectively. Before the age of 12, parents had complete digital access to appointment notifications and could easily monitor their child’s follow-up care, but this possibility changed significantly as the child grew older. Parents began receiving only generic SMS messages indicating that an appointment had been scheduled, without specifying who it was for, where it would take place, or which healthcare professional was involved. This lack of information was especially problematic in families where multiple family members received care, forcing parents to spend time contacting healthcare services to identify the intended recipient of the appointment reminder. The transition at age 12 introduced even more practical complications. For instance, confirmation of consultations was required for travel reimbursements, yet parents were no longer able to access the appointment letters needed for this process. These contradictions, being expected to accompany the child and manage logistics without access to critical information, were described by parents as frustrating and unsustainable. Many parents felt unprepared for this abrupt change when their child reached 12 years of age and criticized the lack of communication and support during this transition. Further complications arose when the child turned 16. When children with complex medical needs reached this age, their parents experienced a sudden and complete loss of access to their child’s medication records and prescriptions. Parents who depended on electronic health records to manage their child’s healthcare described this sudden loss of access as both confusing and distressing, resulting in additional paperwork for them. At the time, many parents were unaware that they needed to apply for formal authorization to regain access.

### Keep the health technology simple

The parents highlighted that a key requirement for the successful adoption of health technology and ensuring that it works well is the implementation of simple, user-friendly solutions that are easy to access. Prioritizing ease of use could significantly enhance user experience and overall performance, and parents described that complicated technology could become a burden. When the parents perceived technology as a burden rather than a support, they avoided or stopped using it. Furthermore, the parents emphasized the importance of being able to use devices that they already owned and minimizing the need for new equipment that would require regular maintenance and take up space in their homes. Smartphones and tablets with accompanying applications (apps) were devices that the parents were familiar with and used daily. Many parents had used apps to log in to electronic health records (such as Helsenorge and HelsaMi) to view their children’s appointments and medical documents. One of the parents indicated that their child with palliative care needs also had diabetes, which increased the number of healthcare services with which they needed to communicate. These parents shared their positive experiences with apps used to communicate medication adjustments with healthcare professionals at the hospital. These apps were connected to their child’s diabetes pump, which sent information about blood sugar levels to healthcare professionals at the hospital. This allowed parents to easily communicate with healthcare professionals and obtain answers on adjustments to the child’s medication and diabetes follow-up. Parents described that this communication mode with healthcare professionals saved time and made follow-up more accessible to both the parents and the child. The parents expressed a preference for simple and accessible apps that allow them to log in when they have time, emphasizing that their daily lives leave little room for complex technology. One parent noted that *“…nothing is better than having an app…*” (P1, FG 5), describing it as *the future* due to the ease of use and intuitive navigation.

The parents stressed that both apps and health technology in general must work well and be updated and reliable to be perceived as supportive rather than disruptive. This was particularly evident in their experiences with video consultations. While all parents had used such consultations, many shared frustrations with technological problems, such as the loss of audio or video. One parent mentioned the following:

*What’s frustrating is when the technology doesn’t work. We’ve had a few video consultations with the specialist hospital that didn’t work properly: either the video or audio failed. We ended up spending 15 min trying to fix it*,* only to switch to a phone call in the end. There have been various issues several times*,* although lately it has been working better* (P2, FG 5).

Parents emphasized that technological challenges could be disruptive, waste time, and shift the focus away from the consultation itself, making them frustrated and dissatisfied with the technological solution.

## Discussion

This study explored Norwegian parents’ perspectives on the use of health technology in home-based PPC, with a particular focus on facilitating communication with healthcare professionals. Parents reported that digital consultations can reduce logistical burdens and enhance communication. However, to ensure meaningful adoption, health technologies must offer user-friendly solutions and be compatible with technological devices that are familiar to parents. Furthermore, electronic healthcare records must provide parents with secure, transparent access to relevant health information about their children, especially during transitions in legal rights related to the child’s care.

Our findings show that video or phone consultations could reduce the disruption of everyday lives for families of children receiving palliative care by offering a less burdensome alternative to in-person hospital visits, particularly when physical examination of the child is not deemed necessary. At the same time, our findings also emphasize that health technology cannot replace the relational aspects of care, underscoring the importance of social influence and the need for trusted healthcare professionals who know the child and family. Nonetheless, previous studies have highlighted that digital consultations can be delivered more flexibly and responsively than in-person visits [18, 20]. Furthermore, these studies found that health technology may enhance the accessibility of healthcare services, reduce logistical burdens for parents, and improve communication between families and healthcare professionals. Similarly, a recent study suggested that families value health technology in home-based PPC when it supports continuity, personalization, and human connection [30]. Consequently, digital consultations should be regarded as a supplement that can enhance accessibility and responsiveness, rather than as a replacement for in-person care.

The parents in our study emphasized the need for health technology to be intuitive, accessible, and compatible with smartphones and tablets with which they were already familiar. Apps giving the opportunity to communicate directly with healthcare professionals were seen as a promising solution due to their simplicity and availability. In families with children facing life-threatening or life-limiting conditions, where time and emotional resources are scarce, simplicity and reliability are not just preferences but are prerequisites for meaningful use [23]. Furthermore, our study found that technical failures during video consultations prevented health technology from being used. In line with the UTAUT framework [23], our findings suggest that for health technology to be meaningfully adopted in home-based PPC, it must be useful, easy to use, socially supported, and backed by reliable infrastructure in the healthcare system. However, if health technology is perceived as too complex or unreliable, parents may avoid or discontinue its use, which undermines its potential benefits [21]. As argued by Mills et al., health technology in PPC must support, not hinder, the caregiving role of families [31]. These insights reinforce the need for end-user-centered design and robust technical support to ensure that health technologies are not only functional but also sustainable in everyday caregiving [18, 32]. A recent systematic review of health technology in PPC found that poor technical performance can weaken trust in health technology and reduce engagement [33]. When a child is receiving PPC with the main goal of supporting the child’s and family’s quality of life [1], this often involves preventing disruptions to the family’s daily routines [2]. These findings highlight that the successful integration of health technology in home-based PPC depends not only on its clinical utility but also on its usability, reliability, and alignment with everyday life. Without careful attention to these factors, even well-intentioned digital solutions risk being underused or abandoned by those who need them the most.

We found that parents in our study have two main needs regarding digital access to sensitive health-related information. On the one hand, they require solutions that enhance coordination and communication with healthcare professionals. On the other hand, they are concerned that unrestricted access for all healthcare providers could lead to data misuse and privacy issues. In addition, parents raise questions about consent, transparency, and control, particularly regarding who decides what information is shared, with whom, and for what purpose. Parents may struggle to navigate these decisions, especially when electronic health records lack clear boundaries or customizable access levels. Previous studies have highlighted that electronic health records must be tailored to the needs of both healthcare professionals and families [20, 34]. The importance of including end-users in the design phase when developing cross-facility electronic healthcare records for PPC has also been highlighted [34]. Balancing access to health-related data with protection of the child’s privacy and security in digital health services requires careful consideration of both practical and ethical dimensions [33]. Concerns about access to a child’s health information, control over it, and its security amid increasing digitalization can significantly influence user behavior [24], especially when shaped by prior experiences of a perceived lack of control over the child’s privacy and health information [33]. By enabling selective sharing of health information and time-limited access, electronic health records could empower parents to safeguard their child’s sensitive information while ensuring sufficient parental oversight, a sense of control, and continuity of their child’s care. In home-based PPC, where families often navigate complex healthcare systems over many years, trust and control over digital information are essential [33]. These additional perspectives highlight the importance of designing health technology solutions that are both user-centered and security-conscious, particularly when dealing with vulnerable populations such as children in need of PPC [30, 33].

Despite the growing interest in health technology, our findings underscore that the field of home-based PPC and the use of health technology remains characterized by fragmented experiences and limited implementation. Parents continue to emphasize basic needs such as usability and intuitive health technology, qualities that should be standard in 2025. While many of the insights presented in this study may appear familiar, it is precisely this slow implementation of health technology in PPC that warrants renewed attention [18, 32]. By focusing on what is still lacking rather than highlighting new innovations, we aim to focus on ongoing gaps in usability, communication, and security, reflecting the current state of practice. These findings indicate that the field has yet to effectively translate existing knowledge into sustainable, user-centered digital solutions.

## Strengths and limitations

One strength of this study is related to our clinical backgrounds, which contributed to deepening the content of the focus groups by allowing us to develop an interview guide with relevant questions. This, in turn, helped us to clarify and enrich the participants’ contributions. However, our familiarity with the field may also have introduced preconceptions or blind spots during the data collection and analysis. To moderate this, we engaged in a reflexive process throughout the study, critically examining our assumptions, roles, and potential biases. This reflexivity enhanced the transparency and trustworthiness of the research [17]. Integrating the UTAUT framework enabled us to interpret our findings within the broader theoretical context of technology acceptance, thereby enhancing the analytical strength of the study.

Dependability was strengthened using the same moderator and consistent main topics in the interview guide across all focus groups. Meanwhile, transferability was supported by the provision of a detailed description of the study context, participant characteristics, data collection, and analytical procedures, along with rich, illustrative excerpts from the participants. This transparency enables researchers and healthcare professionals to assess the relevance of the findings to their own particular context [17].

Another strength of this study is the use of focus groups, which allowed dynamic interaction between participants, enabling rich discussions and the emergence of shared concerns and values [27]. The inclusion of parents representing children with various conditions and care needs in different phases of an illness trajectory, living throughout Norway, also enhanced the credibility of the findings. However, a potential limitation of this work is that some parents participated as couples, while others participated without their partners, which may have influenced the group dynamics and introduced an imbalance in power within the focus groups. Although the inclusion of three fathers in this study is a strength, their minority status within the groups may have contributed to a perceived asymmetrical participation or influence during the discussions. Furthermore, based on the narrow aim of study and the parents’ willingness to share their experiences we deemed that five focus groups generated sufficient information power [35, 36]. One limitation is that one of the focus groups included only three parents. This may have influenced the discussions, potentially omitting certain perspectives and nuances. However, having fewer participants in a focus group can also enhance the opportunity for everyone’s voice to be more prominently heard.

## Conclusions

For children receiving home-based PPC and their families, health technology can contribute to enhanced flexibility in follow-up, reduced burden of traveling to hospitals and consultations, and improved facilitation of everyday life. The value of health technology appears to be contingent upon its usability, access to healthcare systems and electronic health records, and the parents’ ability to maintain control over their child’s health information. Equally important is the need to balance accessibility with security, making sure parents feel confident that security addresses their individual needs.

The findings underscore that healthcare professionals should consider offering digital consultations as a supplement to in-person care when physical examination is unnecessary, thereby improving flexibility and continuity, and reducing logistical strain. Future research should focus on co-designing and evaluating user-friendly applications and secure health record systems with customizable access, particularly during transitions in legal rights. Policy initiatives should address age-related access restrictions and establish clear consent and information-sharing guidelines to balance accessibility with privacy and security. These steps are essential to ensure that health technology supports continuity of care and meets the needs of both the child and family.

## Supplementary Information

Supplementary Material 1.

## Data Availability

The data transcripts generated and analyzed during our study are not publicly available due to privacy restrictions. However, the corresponding author may provide access to data to interested individuals upon reasonable request. It is important to note that obtaining the data will require a reasonable request and permission from the Norwegian Agency for Shared Services in Education and Research.
