# Supplementary material for: Exploring parents’ perspectives on health technology to support communication in home-based pediatric palliative care: a qualitative study
Source: BMC Palliat Care. 2026 Jan 29;25:50. doi: 10.1186/s12904-026-02006-2 (PMC12924306; doi:10.1186/s12904-026-02006-2)
Supplement: Supplementary file 1 — Supplementary Material 1. [file 12904_2026_2006_MOESM1_ESM.docx]

December 2023

**CHIP homeTec - Interview Guide for Focus Groups with Parents**

**Questions:**

1. What do you think of when you hear the term health technology?
   - If the term is unfamiliar, mention other terms like digital solutions, digital technology, and e-health. Provide concrete examples (apps, video solutions, etc.).
2. What experience do you have with using digital solutions in the follow-up of your child?

- Do you currently use any form of digital technology/solutions, video, or applications? If yes, describe.
  - Describe freely regardless of health and the child (e.g., school-home, municipal - specialized health services).
  - How is the child involved in the use of digital technology?
  - What do you find positive/advantageous?
  - What could be different? Barriers?

1. What experience do you have with home-based treatment or follow-up of the child?
   - Outside of hospitals or treatment centers.
   - All types of measures, medical, motor, education, delivery of equipment/assistive devices, medications/pharmacy, mobile teams.
   - If you do not have experience, in what way could this be relevant for your child?
2. How do you view/regard the contributions of digital solutions to the communication/collaboration with various services involved in your child's follow-up?

- What is needed for you to use digital solutions (prerequisites)?

1. What are the most important needs for your family in everyday life?
   - What needs are important: for the child, for siblings, for parents.
   - How can these needs be met?
   - Can digital solutions help meet these needs?
2. Finally, is there anything else you would like to share? Is there a topic you miss, or something else I should have asked about?

**Concluding Questions:**

- Give the floor to the moderator for questions or elaboration of questions.
- The assistant moderator summarizes the main impressions from the interview with the participants.
- How was the interview, and what could have been different?

**Conclusion:**

- Conclude by thanking the parents for participating and then describe the further progress for the study.
- Clarify any practical questions (e.g., reimbursement of travel expenses, etc.).
